# Genetic Basis of Nitrogen-Deficiency-Induced Root Cortical Aerenchyma in Maize Revealed by GWAS and Transcriptome Analysis

**DOI:** 10.3390/plants15010020

**Published:** 2025-12-20

**Authors:** Jianxin Yan, Wenqing Zhang, Qing Tian, Jie Song, Yuzhuo Hou, Haoding Li, Song Cheng, Fang Yang, Hongguang Cai, Yin Wang, Zhe Chen

**Affiliations:** 1 Collage of Resources and Environment, Jilin Agricultural University, Changchun 130118, China; 20241850@mails.jlau.edu.cn (J.Y.); 15352661922@163.com (W.Z.); 19862586083@163.com (Q.T.); songjie010823@163.com (J.S.); houyuzhuo@mails.jlau.edu.cn (Y.H.); haodingli0914@163.com (H.L.); yangfangtum@163.com (F.Y.); 2 Institute of Agricultural Resource and Environment, Jilin Academy of Agricultural Sciences (Northeast Agricultural Research Center of China), Changchun 130033, China; chengsong@jaas.com.cn (S.C.); caihongguang@jaas.com.cn (H.C.)

**Keywords:** root cortical aerenchyma, nitrogen, genome-wide association study, candidate genes

## Abstract

Nitrogen (N) is essential for maize (*Zea mays* L.) productivity, yet its acquisition is limited by the low N uptake efficiency of current varieties. Root cortical aerenchyma (RCA) formation provides a carbon-saving strategy that enhances soil exploration and N acquisition by reducing the metabolic cost of root tissue. However, the genetic basis of RCA formation remains poorly characterized. This study employed an association panel of 295 maize inbred lines to dissect the genetic architecture of RCA formation under low nitrogen (LN) stress. Phenotypic analysis demonstrated that LN stress significantly induced RCA area (RCAA) and proportion (RCAP), with responses ranging from −0.31 to 1.16 mm^2^ for RCAA and −11.34% to 40.18% for RCAP. The non-stiff stalk (NSS) subpopulation exhibited 29.19% higher RCAA under LN than the stiff stalk subgroup. Genome-wide association analysis detected a total of 560 significant SNPs and 810 candidate genes associated with RCA-related traits. Transcriptomic profiling further identified 537 differentially expressed genes between inbred lines with contrasting RCA phenotypes. Integrated GWAS and transcriptomic analysis pinpointed 12 co-localized candidates, subsequently refined to four core genes (*GRMZM2G033570*, *GRMZM2G052422*, *GRMZM2G080603*, and *GRMZM2G472266*), which were implicated in ethylene signaling and stress-responsive root development. Favorable haplotypes of three genes were predominantly distributed in the NSS (25.64–56.00%) and tropical/subtropical (20.51–46.67%) subpopulations. These findings elucidate the genetic basis of LN-responsive RCA formation and provide fundamental resources for marker-assisted breeding of N-efficient maize.

## 1. Introduction

Maize (*Zea mays* L.) is one of the world’s most important cereal crops, accounting for over 15% of global grain production and serving as a vital source of food, feed, and industrial materials [1]. Nitrogen (N) is an essential macronutrient for maize growth and development [2,3]. Compared to other cereal crops, maize requires a larger amount of N to achieve optimal growth and productivity [4,5]. However, N deficiency is widespread and often limits the realization of maize yield potential [6,7]. To enhance maize yield, excessive N fertilizers have been applied, leading to significant resource waste and environmental pollution, partly due to the limited N uptake capacity of maize plants. Improving N use efficiency through breeding or root system regulation represents a promising strategy to mitigate these challenges.

The root system, as the primary organ for nutrient acquisition, exhibits considerable plasticity under low nitrogen stress, remodeling both its architecture and anatomy to enhance soil exploration [8]. Beyond well-characterized morphological adaptations, root anatomical traits, particularly the formation of root cortical aerenchyma (RCA), have been recognized as a key adaptive strategy to abiotic stresses such as hypoxia, waterlogging, and nutrient deficiency, especially nitrogen limitation [9,10]. RCA develops through the generation of air-filled lacunae resulting from programmed cell death in the root cortex [11], a process that significantly reduces the metabolic and structural costs of root tissue [12,13].

The replacement of living cortical parenchyma by air-filled spaces during RCA formation reduces root respiration and nutrient demands, representing a “cheaper root” strategy [10,14,15]. This reduction in tissue construction and maintenance costs enables roots to explore deeper soil layers, thereby enhancing the acquisition of water and nitrogen [16,17,18]. Under nitrogen-deficient conditions, genotypes with enhanced RCA formation demonstrate increased root length, leaf nitrogen content, CO_2_ assimilation, biomass, and yield compared to genotypes with limited RCA [19]. Therefore, screening inbred lines for distinct RCA traits facilitates the breeding of nitrogen-efficient crops.

RCA typically forms through a lysigenous process involving programmed cell death under stress conditions, a response regulated by ethylene, reactive oxygen species (ROS), and transcription factors [11,20,21,22,23,24]. These signaling components may also coordinate other root morphological adaptations, including lateral root development, implying shared regulatory mechanisms in plant responses to edaphic stress [25]. RCA development occurs in two main forms: constitutive, as found in wetland species such as rice and some maize relatives without external stimuli [26,27,28,29], and inducible by stresses. However, distinguishing between these two types remains challenging in practice. Furthermore, under low nitrogen conditions, RCA formation is often assessed in conjunction with other concurrent soil stresses, which complicates the isolation of nitrogen-specific induction [8,24,30]. As a result, the specific role and regulatory mechanisms by which low nitrogen triggers RCA formation remain particularly elusive.

The genetic improvement of RCA began with targeted introgression [31], and subsequent research has progressively elucidated its genetic basis. Early QTL mapping in biparental populations derived from wild teosinte and maize populations such as IBM, NyH, and OhW identified multiple genomic regions associated with RCA formation [32,33,34,35,36,37,38]. Genome-wide association studies further revealed key regulators such as the transcription factor ZmbHLH121 and its upstream causal variant [23], while a large-scale GWAS of 697 inbred lines detected 158 significant SNPs and proposed 64 candidate genes involved in ethylene signaling and sucrose metabolism linked to RCA plasticity [39]. Transcriptome analyses have also provided insights into RCA formation [40]; however, an integrated approach combining GWAS with multi-omics data to identify regulatory candidates for RCA remains lacking. Furthermore, genetic loci specifically associated with RCA induction under low nitrogen stress are still poorly characterized.

This study aims to (i) profile RCA variation under low nitrogen across a maize association panel (295 genotypes) to identify lines with divergent nitrogen response, and (ii) perform GWAS under low and sufficient N conditions while integrating transcriptomics to pinpoint candidate genes. This work provides genetic insights into N-responsive RCA formation and supports breeding of N-efficient maize.

## 2. Results

### 2.1. Phenotypic Variation in Root Cortical Aerenchyma

**Figure 1:**
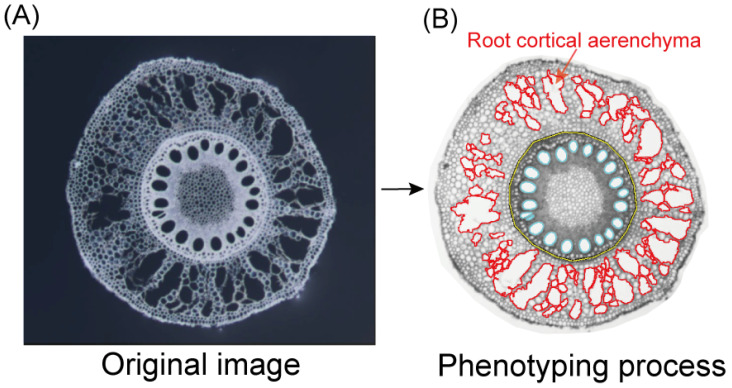
A schematic diagram illustrating the aerenchyma area and its proportion. (**A**) The left image is the original root cross-section. (**B**) The right image shows the analysis process using the RootScan v2.0 software. The red line delineates the root cortical aerenchyma area (RCAA), and the percentage of RCAA relative to the total cortical area represents the root cortical aerenchyma proportion (RCAP).

**Figure 2:**
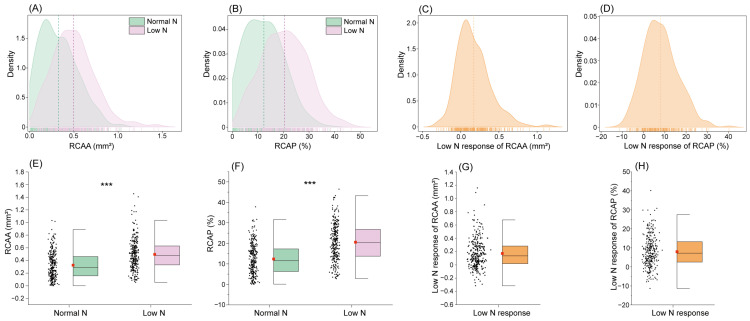
The genetic variation in root cortical aerenchyma under HN and LN conditions. (**A**–**D**) The density plots of RCAA (**A**), RCAP (**B**), LN response of RCAA (**C**) and LN response of RCAP (**D**) in the association population under normal N (NN) and low N (LN) supply conditions. The red point in the boxplots (E-H) and the dotted lines in the density plots (**A**–**D**) indicated the mean values. (**E**–**H**) The boxplots of RCAA (**E**), RCAP (**F**), LN response of RCAA (**G**) and LN response of RCAP (**H**) in the association population under NN and LN supply conditions. The *** in the boxplots (**E**,**F**) indicated the significant differences between normal N and low N treatments (*p* < 0.001). RCAA, RCAP, LN response of RCAA, and LN response of RCAP represent the root cortical aerenchyma area, the proportion of root cortical aerenchyma area in the cortex, the response of RCAA and the response of RCAP to low N supply conditions, respectively. The Low N response is defined as the value under low nitrogen minus the value under high nitrogen for RCAA or RCAP.

The root cortical aerenchyma area (RCAA) and its proportion relative to the cortical area (RCAP) were investigated in an association panel consisting of 295 inbred lines (Figure 1). RCAA and RCAP exhibited approximately normal distributions under both normal N (NN) and low N (LN) conditions, with skewness < 0.75 and kurtosis < 1.14 (Figure 2A,B). Similarly, the LN responses of both traits also showed near-normal distributions (skewness < 1.02, kurtosis < 1.98; Figure 2C,D). Substantial genetic variation was observed for RCAA and RCAP under NN, with variances of 0.21 and 7.49, respectively. Furthermore, these variances increased to 0.24 and 8.88 under LN stress (Table S2). Analysis of variance indicated significant genetic and environmental effects on aerenchyma traits, with broad-sense heritability estimates of 0.51 for RCAA and 0.49 for RCAP (Table S2).

### 2.2. Effects of Low N Stress on Root Cortical Aerenchyma

Root cortical aerenchyma formation was significantly induced under LN stress compared to NN conditions (Figure 2E,F; Table S2). The mean RCAA increased from 0.33 mm^2^ under NN to 0.49 mm^2^ under LN, while the mean RCAP rose from 12.35% (NN) to 20.43% (LN). Furthermore, the LN-response was defined as the value under LN minus the value under HN for RCAA or RCAP. Substantial genetic variation in LN-response was observed across the 295 inbred lines: LN-response of RCAA ranged from −0.31 to 1.16 mm^2^, with a population mean of 0.17 mm^2^, and LN-response of RCAP varied from −11.34% to 40.18%, with a mean increase of 8.08% (Figure 2G,H; Table S2).

### 2.3. Genetic Variations Among Maize Subpopulations

**Figure 3:**
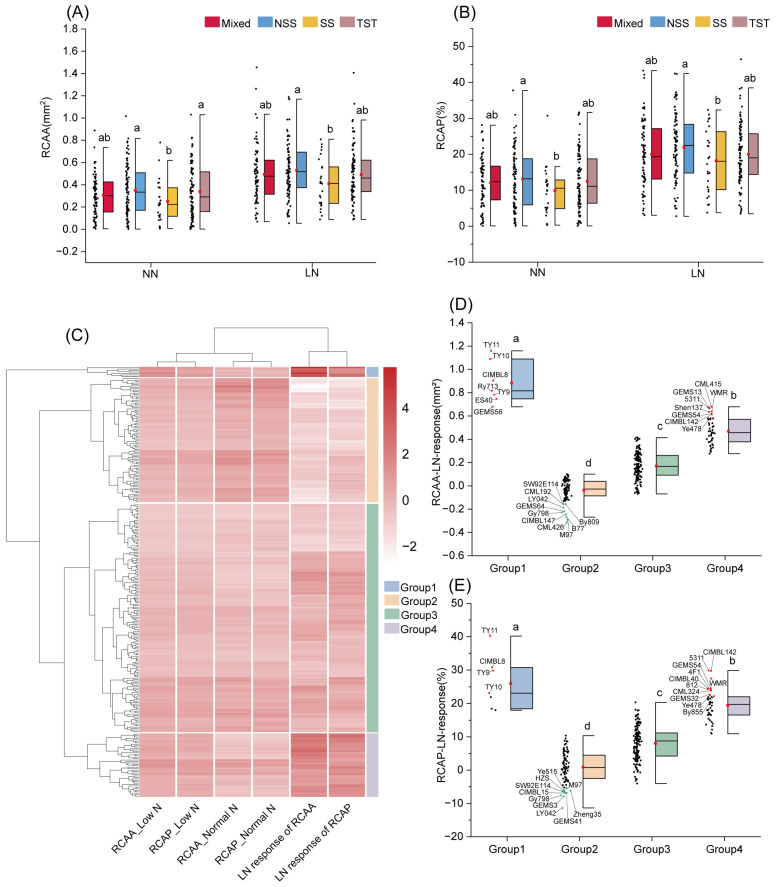
Analysis of subpopulation variation and population clustering for RCAA and RCAP. (**A**,**B**) Variation in RCAA (**A**) and RCAP (**B**) among the Mixed, NSS, SS, and TST subpopulations under normal nitrogen and low N conditions, respectively. Among the subpopulations, SS stands for stiff-stalk, NSS for non-stiff-stalk, TST for tropical and subtropical, and Mixed represents the mixed subpopulation. (**C**) Cluster analysis of the association panel based on their performance (RCAA, RCAP) under normal and low N conditions, as well as their low-N responses. Four clusters were identified: Groups 1, 2, 3, and 4. Red intensity corresponds to higher trait values. (**D**,**E**) Differences in low N response of RCAA (**D**) and RCAP (**E**) among the four clusters identified in (**C**). Red dots with labels mark inbred lines with the highest values; green dots indicate those with the lowest. Different letters represent significant differences between the subpopulations (**A**,**B**) or groups (**D**,**E**) at the *p* < 0.05 level.

Under NN conditions, the NSS and TST subpopulations showed the highest RCAA, with values of 0.34 mm^2^ and 0.35 mm^2^, respectively, both significantly greater than that of the SS subpopulation (0.25 mm^2^). Under LN stress, only the NSS subpopulation maintained significantly higher RCAA, exceeding the SS by 29.19% (Figure 3A). A similar trend was observed for the RCAP, where the NSS subpopulation exhibited significantly higher values than the SS subpopulation under both NN (32.93%) and LN (20.40%) conditions (Figure 3B). In response to LN stress, the NSS subpopulation also displayed the greatest increase in both RCAA and RCAP, rising by 51.16% and 66.92%, respectively (Table S3).

### 2.4. Clustering Analysis and Representative Inbred Lines with Contrasting RCA

Cluster analysis classified the 295 inbred lines into four distinct groups based on their RCAA and RCAP under NN and LN conditions, as well as their LN responses (Figure 3C; Table S4). Significant differences in aerenchyma formation under LN stress were observed among these clusters. Group 1 (*n* = 7) showed the strongest response to LN, with RCAA and RCAP increasing by 0.88 mm^2^ and 26.01%, respectively, values significantly higher than those of all other groups (*p* < 0.05). This was followed by Group 4 (*n* = 44) and Group 3 (*n* = 158). In contrast, Group 2 (*n* = 86) exhibited the weakest LN-response, with a slight decrease in RCAA (−0.03 mm^2^) and a minimal increase in RCAP (0.86%), both significantly lower than the other groups (Figure 3D,E and Figure S1; Table S4).

Based on the clustering analysis and comparisons among inbred lines, we identified representative genotypes with contrasting root aerenchyma phenotypes under LN stress (Figure 3D,E and Figure S1; Table S5). The high-responsive lines, selected from Groups 1 and 4 (e.g., 4F1, TY11, CIMBL8), showed average increases of 0.75 mm^2^ in RCAA and 25.07% in RCAP under LN conditions. In contrast, all these low-responsive lines, derived from Group 2 (e.g., Zheng35, Zheng32, GEMS41), exhibited average changes of −0.22 mm^2^ in RCAA and −4.01% in RCAP. Additionally, a subset of inbred lines distinguished by extreme (high or low) aerenchyma phenotypes was identified, as summarized in Table S5.

### 2.5. Genome-Wide Association Study of RCA Related-Traits

**Figure 4:**
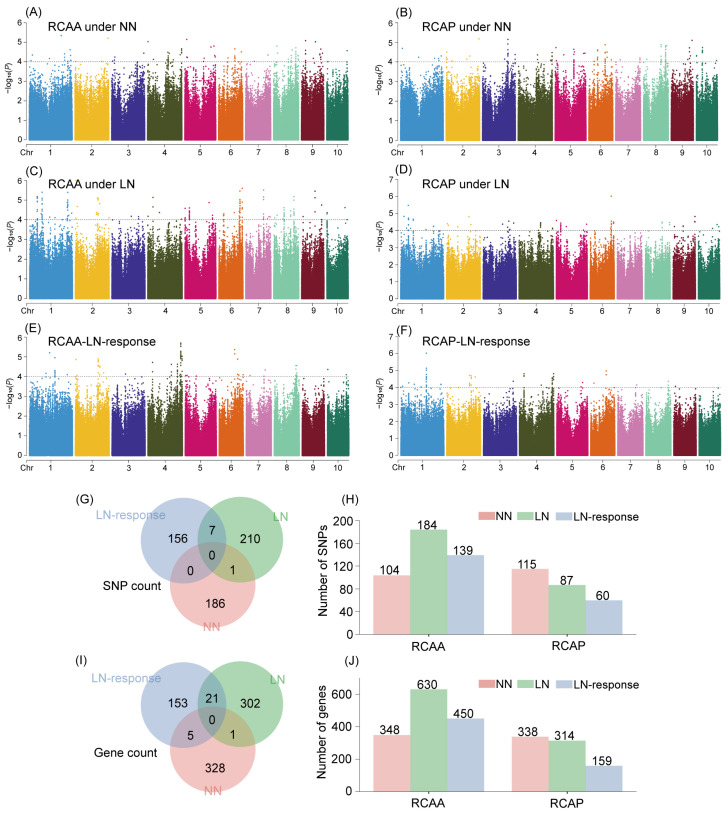
Genome-wide association study of root cortical aerenchyma-related traits. (**A**–**F**) Manhattan plots displaying GWAS results for: (**A**) RCAA under normal nitrogen (NN), (**B**) RCAP under NN, (**C**) RCAA under low nitrogen (LN), (**D**) RCAP under LN, (**E**) LN response of RCAA, and (**F**) LN response of RCAP. The numbers 1–10 on the *x*-axis represent the ten chromosomes of maize. The dashed line indicates the significance threshold of −log_10_(P) = 4.0. (**G**) Venn diagram showing the overlap of significant SNPs among different traits and nitrogen conditions. (**H**) Number of significant SNPs identified for each trait. (**I**) Venn diagram illustrating the overlap of candidate genes. (**J**) Number of candidate genes identified for each trait.

Genome-wide association analysis identified 104, 184, and 139 significant SNPs for RCAA under NN, RCAA under LN, and the LN response of RCAA, respectively. For RCAP, 115, 87, and 60 significant SNPs were detected under NN, LN, and in response to LN, respectively (Figure 4). The phenotypic variation explained by individual SNPs ranged from 6.81% to 21.72% (Table S6). Notably, one SNP was co-located between the NN and LN datasets, and seven SNPs were shared between the LN and LN response datasets.

Candidate gene screening within 50 kb flanking regions of significant SNPs identified 348, 630, 450, 338, 314, and 159 genes for RCAA under NN, RCAA under LN, LN response of RCAA, RCAP under NN, RCAP under LN, and LN response of RCAP, respectively (Figure 4; Table S6). Among these, one gene was co-located between the NN and LN datasets, and 21 genes were co-located between the LN and LN response datasets. Although no SNPs were co-localized between the NN and LN response datasets, five genes were jointly identified due to overlapping 100 kb screening windows surrounding nearby significant SNPs from both datasets. GO enrichment analysis revealed that these genes were enriched in biological processes including cellular process, response to stimulus, and response to stress (Figure S2).

### 2.6. Transcriptomic Analysis of Inbred Lines with Contrasting Low-N Response

**Figure 5:**
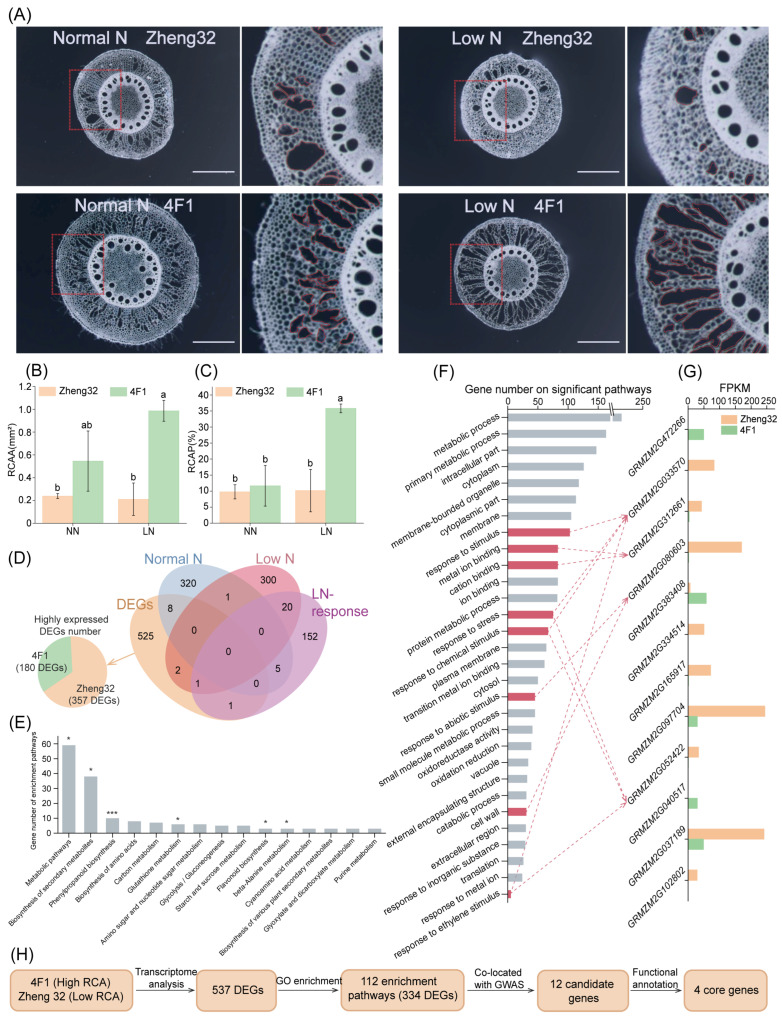
Transcriptome analysis of inbred lines with contrasting aerenchyma phenotypes. (**A**) Images of root cortical aerenchyma (RCA) in Zheng32 (low N-response) and 4F1 (high N-response) under different N treatments. Scale bar = 1 mm. For each pair of images, the left side shows the original root slice, with the area enclosed by a red rectangle enlarged on the right side for display. (**B**,**C**) Comparison of the RCA area (RCAA) (**B**) and RCA proportion (RCAP) (**C**) between Zheng32 and 4F1. Different letters indicate significant differences at the *p* < 0.05 level. (**D**) Transcriptome-derived DEG numbers (pie chart) and their overlap with GWAS candidates from three N conditions (Venn diagram). (**E**) KEGG analysis of DEGs, * and *** indicate significantly enriched metabolic pathways at 0.05 and 0.001 levels, respectively. (**F**) GO enrichment analysis of DEGs. Pathways highlighted in red contain the 12 transcriptome-GWAS co-localized genes. (**G**) Expression of the 12 co-localized genes. Red arrows link genes to their enriched GO pathways. (**H**) Workflow for identifying candidate genes by integrating transcriptome analysis of the phenotypically divergent inbred lines Zheng32 and 4F1 with GWAS co-localization.

Based on previous phenotypic analysis, two inbred lines, 4F1 and Zheng32, exhibiting contrasting aerenchyma formation were selected (Figure 5A). Under LN conditions, 4F1 showed 368.20% greater RCAA and 252.63% higher RCAP compared with Zheng32 (Figure 5B,C). Using previously published transcriptomic data, we identified 537 differentially expressed genes (DEGs) between these two lines, among which 180 were upregulated in 4F1 and 357 were upregulated in Zheng32 (Figure 5D). KEGG analysis revealed that the DEGs were enriched in metabolic pathways related to plant stress resistance and environmental interaction, such as the Biosynthesis of secondary metabolites and Phenylpropanoid biosynthesis (Figure 5E). GO enrichment analysis revealed that 334 DEGs were significantly enriched in 112 biological processes, including response to stimulus and response to stress (Figure 5F).

A comparative analysis identified 12 genes that overlapped between the DEG set and the GWAS candidates (Figure 5G). Of these, three were upregulated in 4F1 and nine in Zheng32 (Figure 5G). Four of the 12 overlapping genes were associated with the top 30 most significantly enriched GO terms. These 12 co-localized genes, identified by integrating GWAS and transcriptome analysis, were targeted for subsequent refinement through gene annotation and haplotype analysis (Figure 5H).

### 2.7. Haplotype Analysis and Functional Annotation of Candidate Genes

**Figure 6:**
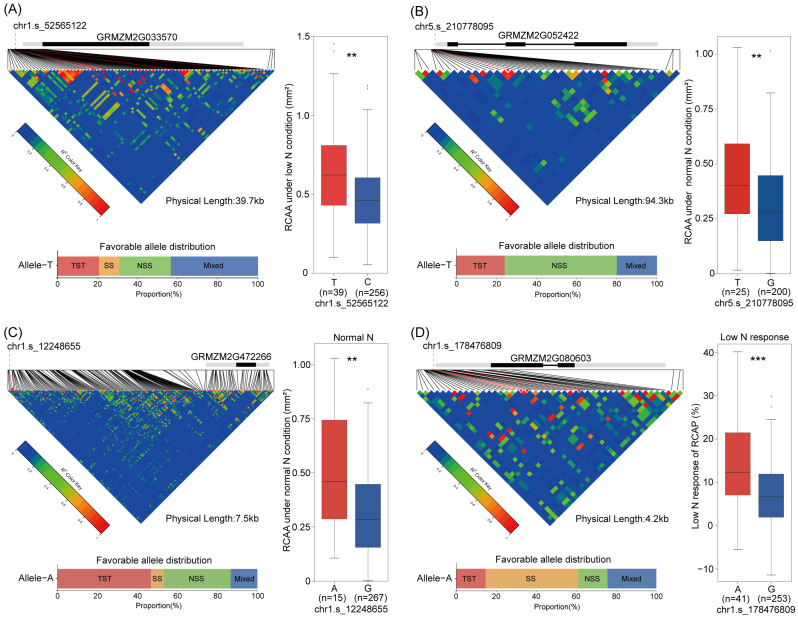
Linkage disequilibrium and haplotype analysis of four core candidate genes. (**A**–**D**) Linkage disequilibrium and haplotype analysis of *GRMZM2G033570* (**A**), *GRMZM2G052422* (**B**), *GRMZM2G472266* (**C**), and *GRMZM2G080603* (**D**). In each panel, the left upper part shows the linkage disequilibrium heat map, with red color intensity indicating the strength of linkage, and the vertical red line marks the position of the most significantly associated SNP. The right side of each panel displays box plots comparing phenotypic traits between haplotypes defined by the most significant SNP; ** and *** indicate significant differences between haplotypes at *p* < 0.01 and *p* < 0.001 level, respectively. The bottom of each panel shows the distribution frequency of the favorable allele across the four subpopulations.

Haplotype analysis was performed for the 12 co-located genes identified through integrated GWAS and transcriptomics; four of these genes were significantly associated with root aerenchyma formation (Figure 6). For *GRMZM2G033570*, lines carrying haplotype T (*n* = 39) exhibited 38.89% greater RCAA under LN conditions than those with haplotype C (*n* = 256). Similarly, for *GRMZM2G052422*, haplotype T (*n* = 25) increased RCAA by 35.57% under NN compared to haplotype G (*n* = 200). *GRMZM2G472266* haplotype A (*n* = 15) conferred a 72.88% advantage in RCAA under NN over haplotype G (*n* = 267), while *GRMZM2G080603* haplotype A (*n* = 41) enhanced the LN response of RCAP by 97.49% compared to haplotype G (*n* = 253).

The favorable alleles of *GRMZM2G033570*, *GRMZM2G052422*, and *GRMZM2G472266* were more frequent in the NSS (25.64–56.00%) and TST (20.51–46.67%) subpopulations than in SS (0–10.26%). In contrast, the favorable allele of *GRMZM2G080603* occurred at a higher frequency in SS (46.34%) than in the other subpopulations (14.63–24.39%). In addition, the 26 inbred lines, which combined two favorable haplotypes, exhibited significantly higher RCAA and RCAP—by approximately 30.51–41.60% under normal nitrogen conditions and 26.97–53.88% under low nitrogen conditions—compared to inbred lines carrying no or only one favorable allele (Figure S3). Functional annotations associated these genes with stress-responsive root development. *GRMZM2G052422* participates in ethylene-mediated signaling that promotes root aerenchyma formation. *GRMZM2G033570* functions in the ethylene signaling pathway aiding root adaptation to compacted soil. *GRMZM2G080603* is a candidate gene for lateral root development under low phosphorus stress, while *GRMZM2G472266* is a drought-responsive gene with an uncharacterized function in roots.

## 3. Discussion

### 3.1. The Phenotypic Variance of RCA in Response to Low N Stress

Under N deficiency, the formation of root cortical aerenchyma (RCA) reduces root metabolic costs by decreasing respiration and nutrient demands, thereby promoting deeper root growth and enhancing N uptake efficiency [12,15]. Given these benefits, RCA is considered a promising target for breeding N-efficient crops. Previous studies have reported substantial genotypic variation in RCA formation, which we have also observed [9,16,41]. More importantly, this study presents the first systematic quantification of RCA plasticity under low N stress in a large association population. Our results showed that the LN response ranged approximately between −0.31 to 1.16 mm^2^ for RCAA and −11.34% to 40.18% for RCAP, respectively, with significant genotypic variation. Furthermore, we identified inbred lines exhibiting extreme RCA plasticity under low-N stress, including high-response types such as 4F1 and TY11, and low-response types such as Zheng32 and Zheng35 (Figure 3; Table S5). These lines represent valuable genetic resources for breeding N-efficient maize with reduced root metabolic costs.

In previous studies, root morphology traits in maize have often shown a moderate heritability, and root cortical aerenchyma exhibits even lower heritability, primarily due to its high sensitivity to environmental stress [42,43,44]. In this study, root cortical aerenchyma formation was induced by low N stress, yet significant varietal differences were observed. The moderate heritability (≈0.5) observed here is driven by a substantial increase in environmental variance and pronounced genotype-by-environment (G × E) interactions specific to N stress (Table S2). Consequently, the genetic architecture underlying root aerenchyma plasticity under low N fundamentally differs from that under normal conditions. This strongly supports the perspective that the low N response constitutes a distinct genetic trait in this study.

Previous studies reported that the SS subpopulation generally forms thinner and longer roots under low N stress, whereas NSS and TST subpopulations develop thicker and shorter roots [45,46]. In this study, we found that NSS and TST subpopulations typically form more extensive aerenchyma under LN stress, whereas the SS subpopulation shows less RCA and a weaker plastic response. This suggests divergent metabolic strategies for carbon conservation: the SS subpopulation may minimize carbon costs primarily through reduced root diameter, whereas the NSS/TST may rely more on RCA formation to lower respiration demands [13,47]. These findings point to a potential coordination between root anatomical and morphological adaptations to LN stress. Furthermore, these differences in low-N root plasticity responses indicate significant differentiation among subpopulations during maize evolution. From a domestication perspective, TST represents a tropical subpopulation, whereas NSS and SS are largely temperate, suggesting that root traits were also selected during the divergence between tropical and temperate germplasm. Moreover, the significant differences in RCA between the non-stiff-stalk (NSS) and stiff-stalk (SS) subpopulations imply that root aerenchyma formation may have been selected in parallel with aboveground traits such as stalk architecture [48,49]. These observations collectively highlight the importance of further research into its genetic and evolutionary basis.

### 3.2. Integrating GWAS and Transcriptomics to Uncover the Genetic Basis of RCA

Over the past few decades, numerous genetic studies have been conducted on root cortical aerenchyma (RCA) and have identified regulatory loci and candidate genes [32,33,34,35]. However, these studies were largely based on absolute measurements of RCA area under single or stress conditions. It is established that RCA exhibits a response to low nitrogen, characterized by the formation of specific lysigenous aerenchyma [11,12]. The genetic basis of RCA plasticity in response to low N, particularly regarding the extent of aerenchyma formation induced by low N, remains unclear. In this study, we performed genome-wide association mapping under both normal and low nitrogen conditions, with a specific focus on the low nitrogen response. Our approach helped to clarify the genetic mechanisms underlying RCA plasticity under low nitrogen stress and led to the identification of 153 candidate genes. These findings offer deeper insights into the genetic foundation of root phenotypic plasticity [50].

Furthermore, although previous QTL mapping studies have identified several key loci associated with RCA, their limited resolution has impeded the identification of candidate genes [32,33,34,35,36,38,44]. While genome-wide association studies (GWAS) offer better mapping efficiency, they frequently generate candidate genes with high false-positive rates [51]. Integrating forward genetic mapping with multi-omics approaches has proven to be an effective strategy for identifying candidate genes [52]. In this study, we combined GWAS with transcriptomic analysis of inbred lines showing contrasting RCA phenotypes. This integrated strategy efficiently refined the candidate gene list, resulting in the identification of 12 core candidate genes (Figure 5; Table S6). Notably, several of these genes were co-located with chromosomal regions previously identified as hotspots for root traits [53]. For instance, *GRMZM2G097704* was located within the known hotspot bin 5.05–5.06, *GRMZM2G052422* was mapped to bin 5.07, and *GRMZM2G102802* co-localized with bin 9.06. Meanwhile, the previous research on maize genes responding to low-nitrogen signals is also positioned in this paper. The *GRMZM2G037368* and *AC149818.2_FG009* identified through GWAS have been shown to be involved in low-nitrogen signaling, and they regulate the formation of maize seminal roots and lateral roots, respectively [54,55,56]. These findings suggest that these candidate genes represent robust genetic signals and may serve as valuable resources for future research on maize root system architecture.

### 3.3. The Possible Molecular Regulation of Candidate Genes

Through Gene Ontology analysis, it was revealed that the gene sets derived from both GWAS and transcriptomic studies are significantly enriched in biological processes such as response to stimulus and response to stress. By integrating GWAS and transcriptomic studies, we identified 12 core candidate genes, and further functional annotation refined this list to four key genes. Among them, *GRMZM2G033570* and *GRMZM2G052422* are associated with ethylene-mediated regulation of plant development [57,58,59,60,61,62,63]. Since ethylene is a crucial hormone controlling the formation of RCA, these two genes are strong candidates as core regulators mediating RCA plasticity in response to low N conditions. Additionally, *GRMZM2G080603* has been implicated in low-phosphorus stress tolerance and root development [64]. Its homologous gene in Arabidopsis participates in ABA signaling, suggesting its potential role in modulating RCA formation through stress-related signaling pathways [65]. *GRMZM2G472266* has been mentioned to be related to the regulation of root shoot ratio under drought stress, and is highly likely to be involved in RCA regulation [66]. For subsequent research, the known ethylene-related genes require in-depth investigation to elucidate their precise regulatory mechanisms in low nitrogen adaptation or RCA formation, while the functions of other less-characterized genes need to be clarified through molecular biology validation using transgenic plants and mutant materials (Table 1).

Root architecture development and its disparities in response to low N exist among maize subpopulations [67,68]. As mentioned above, such differences among subpopulations were also observed for RCA in this study. To uncover the genetic basis underlying these differences, we first conducted haplotype analysis of candidate genes and further examined the distribution of favorable alleles across different subpopulations. We observed significant subpopulation-specific distributions of favorable alleles for *GRMZM2G052422*, *GRMZM2G033570*, and *GRMZM2G472266*, with their favorable alleles being more prevalent in the NSS subpopulation (Figure 6). Furthermore, in the association panel, we found that 26 genotypes carrying favorable haplotypes across different genes exhibited enhanced RCA (Figure S3). These findings provide insights into the genetic basis underlying the differential RCA and contrasting low-N responses among subpopulation germplasms, offering valuable genetic resources for breeding N-efficient maize varieties. We look forward to further in-depth domestication analysis and molecular biology validation of these important candidate genes and their favorable allelic variations.

## 4. Materials and Methods

### 4.1. Plant Materials

A total of 387 genotypes from the panel established by [69] were initially planted. From these, 295 maize inbred lines were retained for the study after applying the following exclusion criteria: (1) poor root section quality caused by shrinkage, disease, or insect damage; (2) normal growth under only one treatment; or (3) failure in seed propagation. The final association panel consisted of four subpopulations: stiff stalk (SS, *n* = 26), non-stiff stalk (NSS, *n* = 95), tropical/subtropical (TST, *n* = 95), and mixed (*n* = 79). Detailed pedigree and subpopulation information are provided in Table S1.

### 4.2. Field Experiment Design

The field trial was conducted during the 2023 growing season at the Experimental Station of Jilin Agricultural University (43°8′ N, 125°4′ E). The soil before sowing in the 0–30 cm layer contained alkaline-hydrolyzable N at 85.95 mg kg^−1^, available potassium at 93.12 mg kg^−1^, available phosphorus at 33.91 mg kg^−1^, organic matter at 19.37 g kg^−1^, and had a pH of 6.76. Basal fertilizer (containing phosphorus at 21.8 kg ha^−1^ and potassium at 41 kg ha^−1^) was applied before sowing. All management practices adhered to local agricultural standards.

The experiment comprised two N input treatments: normal N (NN, 180 kg N ha^−1^) and low N (LN, no fertilizer N). For each treatment, an augmented α design was adopted following [70]. The trial included four replicates per N treatment, with each replicate divided into 295 single-row plots. Each plot measured 2 m in length, with row spacing of 0.6 m and plant spacing of 0.2 m, and contained one genotype. At the V4 (four-fully expanded leaves) stage, three plants per plot were harvested for root morphology analysis in a separate study. The remaining plants were retained for the assessment of root anatomy in this study at the VT (tasseling) stage.

### 4.3. Root Sampling and Phenotype

Root systems were sampled at the VT (tasseling) stage by excavating the three central plants per row with a shovel. Subsequently, a 2 cm segment was collected from the fourth whorl of crown roots at 3–5 cm from the stem base. These root segments were immediately fixed in FAA solution for anatomical sectioning according to the method of [44].

Root cross-sections were prepared according to established methods [44,71]. Briefly, 2 cm root segments were manually sectioned and observed under a light microscope (Olympus Corporation, CX31, Tokyo, Japan). One section of uniform thickness per plant was selected for imaging under an optical microscope. The resulting JPEG images were analyzed using RootScan2.jar software to quantify the root cortical aerenchyma area (RCAA) and its proportion relative to the cortex (RCAP) [37].

To quantify the LN response of RCAA and RCAP, which also reflects the induction of lysigenous aerenchyma under N stress, we used the following calculation:

(1) LN response = Value under LN − Value under NN

### 4.4. Phenotypic Variance Analysis

Variance component, heritability, and best linear unbiased estimator (BLUE) were calculated based on the following model:

(2) y=μ+G+N+G×N+Rep(N)+ε

where μ was overall mean, G was the genotype effect, N was the N treatment effect, G × N was the genotype-by-N interaction effect, and Rep (N) was the replication effect within each N treatment, and ε was the error. All factors were considered random for estimating variance components. For the calculation of BLUEs under each N treatment (normal-N and low-N) separately, the genotypic effect (G) was treated as fixed, and the model excluded the N and G × N effects.

Phenotypic evaluation was conducted using the best linear unbiased estimates (BLUEs) for each trait. Descriptive statistics (mean, maximum, and minimum) were computed with SPSS 21.0. Data distribution normality was assessed based on skewness and kurtosis, with normality defined as absolute skewness < 3 and absolute kurtosis < 10 [45]. To compare root traits among subpopulations within the same N treatment, a one-way ANOVA followed by Duncan’s multiple range test was performed. The results were visualized using boxplots generated with the ‘ggplot2’ package v3.5.1 in R v4.4.1.

### 4.5. Broad-Sense Heritability

Broad-sense heritability (*H*^2^) was estimated using the following formula [72]:

(3) $$H^{2}\;=\;\frac{\sigma_{G}^{2}}{\sigma_{G\;}^{2}+\;\frac{\sigma_{G\times E}^{2}}{n}\;+\;\frac{\sigma_{\varepsilon }^{2}}{\mathrm{nr}}}$$

where $\sigma_{G}^{2}$, $\sigma_{G\times E}^{2}$, and $\sigma_{\varepsilon }^{2}$ represented the genotypic variance, genotype-by-environment interaction variance, and error variance, respectively, as obtained from model (2) across N treatments; n denoted the number of N treatments (2 in this study), and r referred to the number of replicates (4 in this study). All analyses were conducted using the ‘sommer’ package v4.4.3 in R.4.1.1 [73].

### 4.6. Clustering Analysis

The BLUE value for each genotype was standardized using the following formula:

(4) $$\mathrm{Yi}\;=\;\frac{(\mathrm{Xi}\;-\;\mathrm{mean}(X))}{\mathrm{mean}(X)}$$

where Xi was the original BLUE value of the aerenchyma trait for a given genotype, mean(X) was the mean value across all 295 genotypes, and Yi was the resulting standardized value. These standardized values were then utilized for cluster analysis [74].

Based on the standardization matrix, the Euclidean distance between all pairs of the 295 inbred lines was computed to construct a phenotypic clustering tree. Data visualization, including the clustering tree and heatmaps of the standardized trait values, was performed using the ‘pheatmap’ package v1.0.12 in R v4.4.1.

### 4.7. Genome-Wide Association Study

A genome-wide association study (GWAS) was conducted using Tassel v5.2.77 with a compressed mixed linear model (CMLM). The model included 1,253,814 SNP markers and accounted for population structure (Q) and kinship (K) as covariates, with the BLUEs of root traits serving as phenotypic inputs [75]. A significance threshold of −log_10_(P) ≥ 4.0 was applied to balance false positives and false negatives, as recommended by [76]. Resulting associations were visualized in Manhattan plots generated with the ‘cmplot’ package v4.5.1 in R v4.4.1. Candidate genes were defined as those located within 50 kb upstream and downstream of each significant SNP, based on annotations from the B73 RefGen_v2 genome.

### 4.8. Transcriptome Analysis of Inbred Lines with Contrasting Phenotypes

To identify candidate genes associated with root cortical aerenchyma formation, we analyzed a published transcriptomic dataset from two inbred lines with contrasting aerenchyma phenotypes: 4F1 (high-aerenchyma) and Zheng32 (low-aerenchyma) [76]. Differentially expressed genes (DEGs) were identified using the criteria of |log_2_FC| > 1.0 and FDR < 0.01 [77]. These DEGs were subsequently subjected to Gene Ontology (GO) enrichment analysis. Gene expression patterns were visualized in a heatmap using the ‘pheatmap’ package v1.0.12 in R v4.4.1. Finally, transcriptomic candidates were integrated with GWAS loci to define a high-confidence gene list.

### 4.9. Linkage Disequilibrium, Haplotype Analysis and Annotations of Candidate Genes

For the high-confidence candidate genes identified through integrated GWAS and transcriptome analysis, linkage disequilibrium (LD) blocks were defined using TASSEL 5.0 and visualized with the ‘LDheatmap’ package v1.0.6 in R v4.4.1. Haplotype groups were constructed based on the allelic variation in the most significantly associated SNPs, and their effects on phenotypes were assessed by one-way ANOVA and presented using boxplots generated with the ‘ggplot2’ package v3.5.1 in R v4.4.1. Functional annotations for these candidate genes were obtained from MaizeGDB (https://www.maizegdb.org/).

## 5. Conclusions

In this study, phenotypic characterization of root cortical aerenchyma (RCA) across 295 maize inbred lines demonstrated significant induction of RCA under low nitrogen (LN) stress at the population level, coupled with substantial genotypic variation in LN responsiveness. The NSS subpopulation exhibited superior RCA plasticity, and distinct high- and low-responsive inbred lines were identified, offering valuable germplasm for root-oriented breeding. Through integrated GWAS and transcriptomic profiling, we identified 12 candidate genes, subsequently refined to four core candidates via haplotype analysis, GO enrichment, and functional annotation. These genes are associated with stress response pathways, including ethylene signaling, and root development. Their favorable haplotypes showed significant subpopulation divergence, being predominantly distributed in the NSS and TST groups. Collectively, these findings provide fundamental genetic resources and functional candidates for marker-assisted selection, facilitating the development of nitrogen-efficient maize varieties through optimized root system architecture.

## Figures and Tables

**Table 1:** Functional annotation of core candidate genes putatively involved in root response to low N.

Gene ID	Gene Names	Sources ^a^	Genes Annotation of Maize and Homologs ^b^	Reference ^c^
*GRMZM2G052422*	*acco35—1-aminocyclopropane-1-carboxylate oxidase35*	Co-localization of DEGs and HN	Ethylene-forming enzyme (*AT1G05010.1*, *Arabidopsis thaliana*); 1-aminocyclopropane-1-carboxylate oxidase protein (*LOC_Os02g53180.2*, *Oryza sativa*); Participating in the ethylene-mediated pathways that promote root development, including cortical aerenchyma formation (*Zea mays*)	[57,58,59,60,61]
*GRMZM2G033570*	*eil3—ethylene insensitive-like3*	Co-localization of DEGs and LN	Ethylene-insensitive 3 family protein (*AT3G20770.1*, *Arabidopsis thaliana*); Ethylene-insensitive 3, putative, expressed (*LOC_Os03g20790.1*, *Oryza sativa*); Involving in the ethylene signaling pathway underlying root adaptation to compacted soil conditions (*Zea mays*)	[57,62,63]
*GRMZM2G080603*	*grp1—glycine-rich protein1*	Co-localization of DEGs and LN response	Cold, circadian rhythm, and RNA binding 2 (*AT2G21660.1*, *Arabidopsis*); RNA recognition motif containing protein, expressed (*LOC_Os12g43600.1*, *Oryza sativa*); Candidate gene of lateral roots development under low phosphorus stress (*Zea mays*)	[64,65]
*GRMZM2G472266*	*umc1723a*	Co-localization of DEGs and HN	Unknown (*AT3G22530.1*, *Arabidopsis thaliana*); Expressed protein (*LOC_Os03g06390.1*, *Oryza sativa*); Drought-responsive gene expression in roots with uncharacterized function (*Zea mays*)	[66]

Note: ^a^ “Sources” refers to the origin of the gene mapping and transcript analysis information. ^b^ The elucidated function of this gene in maize, as well as its homologous genes in *Oryza sativa* and *Arabidopsis* and functional annotations, are described separately. ^c^ References pertaining to the function of the candidate gene in maize.

## Data Availability

Data are available upon request.

## Supplementary Materials

The following supporting information can be downloaded at: https://www.mdpi.com/article/10.3390/plants15010020/s1. Figure S1: RCAA and RCAP variations under clustering grouping; Figure S2: Significantly enriched GO terms of the three categories of candidate genes (Figure 4I) under normal N, low N, and low N response conditions. Figure S3: Distribution of favorable allele variations and phenotypic characteristics of RCAA and RCAP. Table S1: Information of the association mapping panel consisting of 295 maize inbred lines; Table S2: Phenotypic variances of maize root anatomical traits in normal nitrogen, low nitrogen, and low nitrogen response datasets; Table S3: Differences in root anatomical traits of maize under different subgroups; Table S4: Differences in root anatomical traits of maize under different clustering groups; Table S5: Extreme inbred lines of RCAA traits under low nitrogen stress; Table S6: Significant SNP and candidate genes detected in genome wide association study; Table S7: GO-enrichment analysis of candidate genes detected in differential expression genes (DEGs), normal N, low N and low N response datasets; Table S8: Phenotypic differences among haplotypes of core candidate genes in this study.
