# Supplementary material for: Genetic Basis of Nitrogen-Deficiency-Induced Root Cortical Aerenchyma in Maize Revealed by GWAS and Transcriptome Analysis
Source: Plants (Basel). 2025 Dec 20;15(1):20. doi: 10.3390/plants15010020 (PMC12787405; doi:10.3390/plants15010020)
Supplement: Supplementary file 1 [file plants-15-00020-s001.zip › Figure S1.pdf]

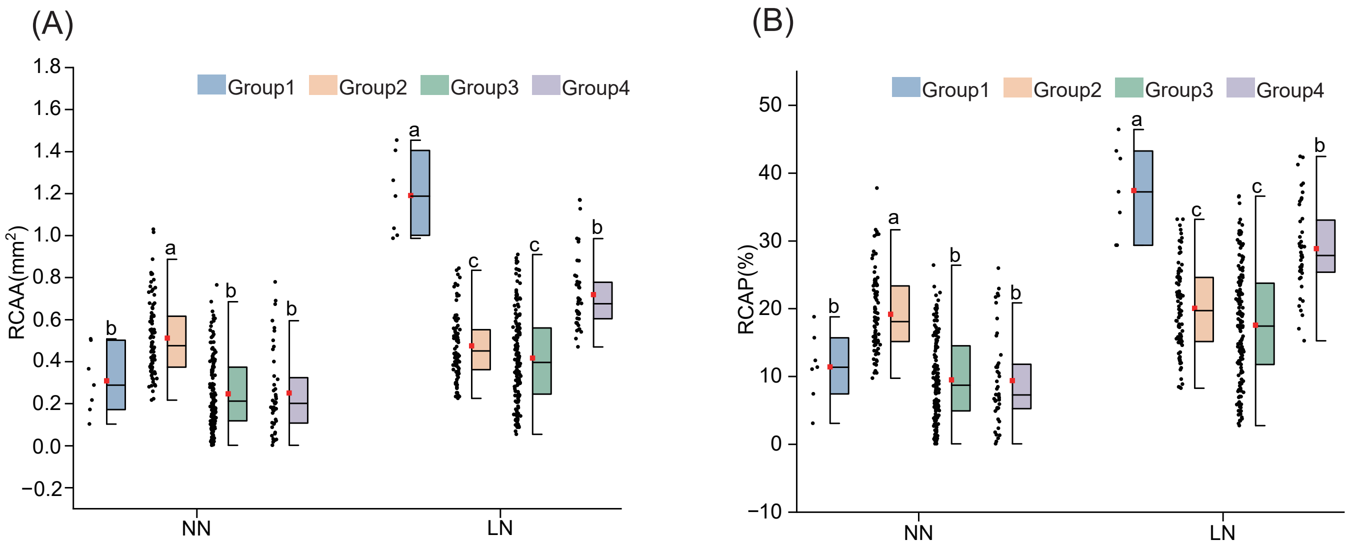

Figures S1. RCAA and RCAP variations under clustering grouping. (A-B) Changes in RCAA (A) and RCAP (B) among the four clusters—Group 1, Group 2, Group 3, and Group 4—identified in (Figure. 3C) under normal nitrogen and low nitrogen conditions, respec-tively. The red points in the boxes represent the mean values, different letters indicate significant differences at the $P < 0.05$ level.
