# Supplementary material for: Genetic Basis of Nitrogen-Deficiency-Induced Root Cortical Aerenchyma in Maize Revealed by GWAS and Transcriptome Analysis
Source: Plants (Basel). 2025 Dec 20;15(1):20. doi: 10.3390/plants15010020 (PMC12787405; doi:10.3390/plants15010020)
Supplement: Supplementary file 1 [file plants-15-00020-s001.zip › Figure S2.pdf]

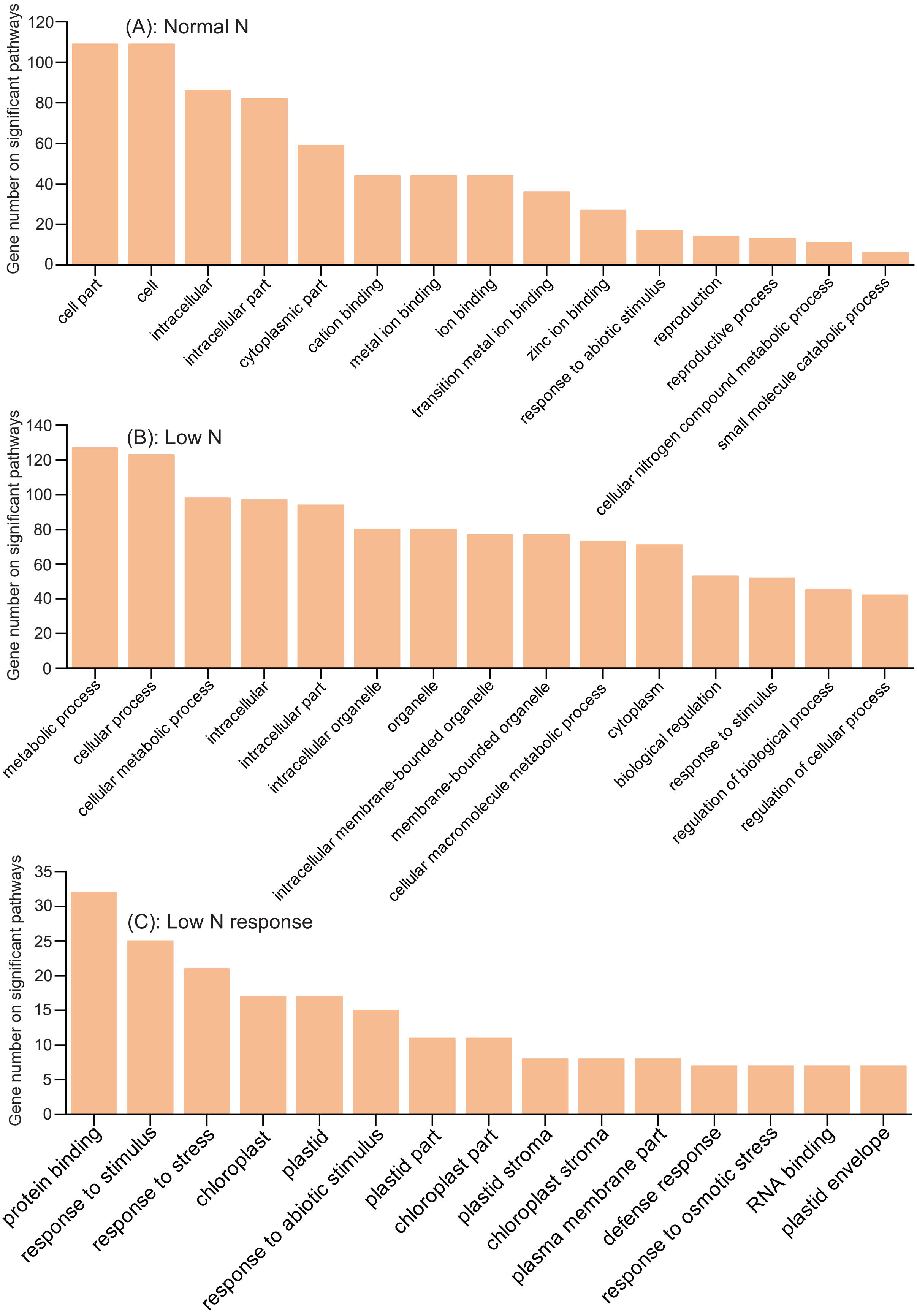

Figure S2. Significantly enriched GO terms of the three categories of candidate genes (Figure 4I) under normal N, low N, and low N response conditions. (A) Top 15 significantly enriched GO terms of candidate genes under normal N conditions. (B) Top 15 significantly enriched GO terms of candidate genes under low N conditions. (C) Top 15 significantly enriched GO terms of candidate genes under low N response conditions.
