# Supplementary material for: Genetic Basis of Nitrogen-Deficiency-Induced Root Cortical Aerenchyma in Maize Revealed by GWAS and Transcriptome Analysis
Source: Plants (Basel). 2025 Dec 20;15(1):20. doi: 10.3390/plants15010020 (PMC12787405; doi:10.3390/plants15010020)
Supplement: Supplementary file 1 [file plants-15-00020-s001.zip › Figure S3.pdf]

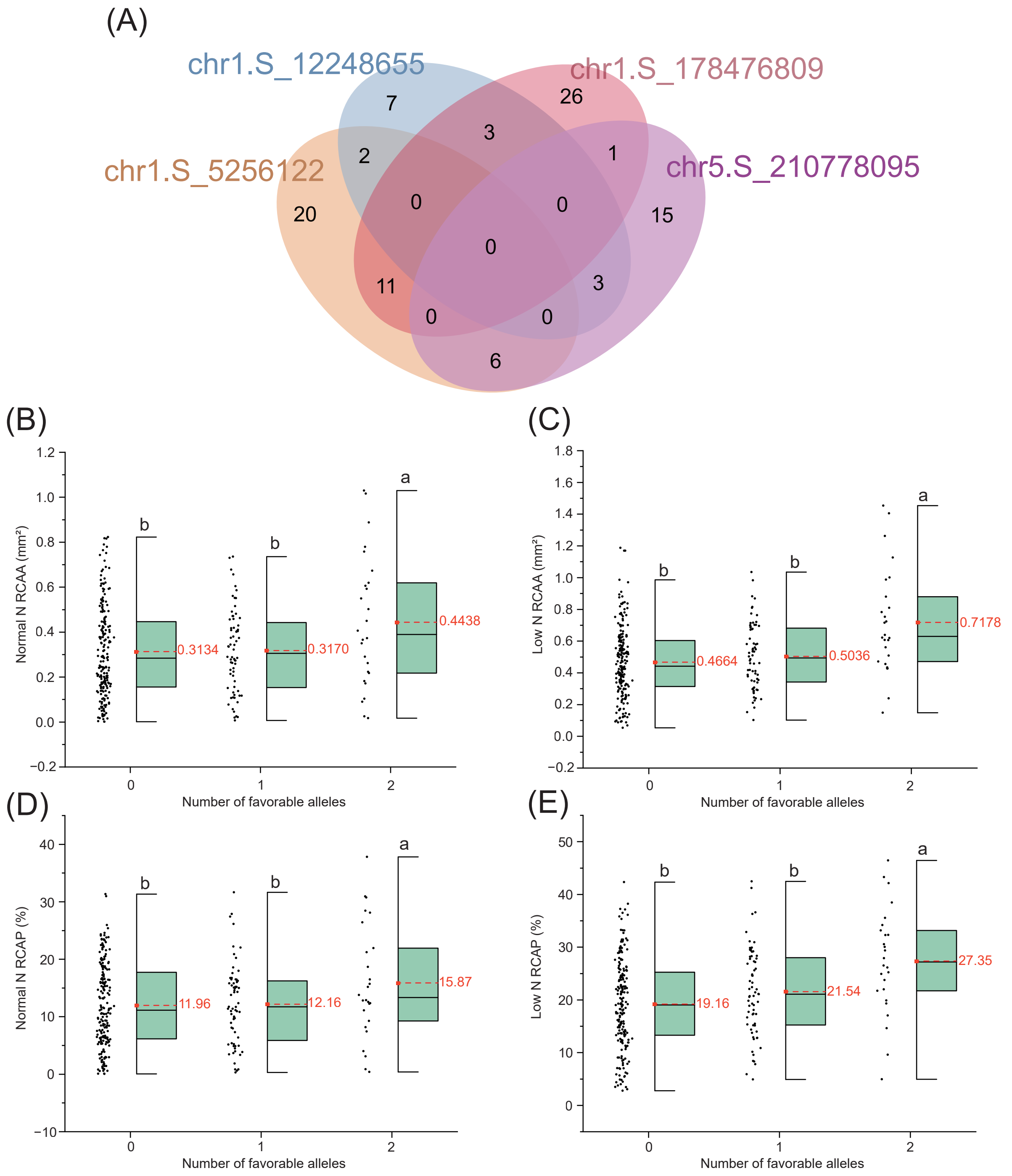

Figure S3. Distribution of favorable allele variations and phenotypic characteristics of RCAA and RCAP. (A) Distribution of the favorable allele at the four SNP loci across varieties in Figure 6. Changes in normal N RCAA(B), low N RCAA(C), normal N RCAP(D), and low N RCAP(E) under different favorable allele additive effects. Different letters indicate significant differences at the P < 0.05 level.
